# Quality of life of patients with Parkinson's disease: a comparison between preoperative and postoperative states among those who were treated with deep brain stimulation

**DOI:** 10.1590/0004-282X-ANP-2021-0048

**Published:** 2022-03-14

**Authors:** Maria Eduarda Turczyn de Lucca, Jhulia Farinha Maffini, Mariana Guerrini Grassi, Amanda Elias Abdala, Renato Mitsunori Nisihara, Alexandre Novicki Francisco, Marina Farah, Tatiana von Hertwig Fernandes de Oliveira Kumer

**Affiliations:** 1 Universidade Positivo, Curitiba PR, Brazil.; 2 Hospital Universitário Cajuru, Curitiba PR, Brazil.

**Keywords:** Parkinson Disease, Deep Brain Stimulation, Quality of Life, Doença de Parkinson, Estimulação Encefálica Profunda, Qualidade de Vida

## Abstract

**Background:**

Deep brain stimulation (DBS) is a well-established procedure for treating Parkinson's disease (PD). Although its mechanisms of action are still unclear, improvements in motor symptoms and reductions in medication side effects can be achieved for a significant proportion of patients, with consequent enhancement of quality of life.

**Objective:**

To investigate the impact of DBS on the quality of life of PD patients.

**Methods:**

This was a retrospective longitudinal study with collection of historical data in a neurosurgery center, from June 2019 to December 2020. The sample was obtained according to convenience, and the Parkinson's Disease Questionnaire (PDQ-39), Unified Parkinson's Disease Rating Scale (UPDRS) III and IV, Trail-Making Test and Verbal Fluency Test were used.

**Results:**

Data were collected from 17 patients (13 with subthalamic nucleus DBS and 4 with globus pallidus pars interna DBS). Significant improvement (p=0.008) on the UPDRS III was observed in comparing the preoperative without DBS with the postoperative with DBS. About 47.0% of the patients showed post-surgical improvement in QoL (p=0.29). Thirteen patients were able to complete part A of the Trail-Making Test and four of these also completed part B. Almost 60% of the patients scored sufficiently on the semantic test, whereas only 11.8% scored sufficiently on the orthographic evaluation. No association between implant site and test performance could be traced.

**Conclusions:**

Improvements in quality of life and motor function were observed in the majority of the patients enrolled. Despite the limitations of this study, DBS strongly benefits a significant proportion of PD patients when well indicated.

## RESUMO

**Antecedentes:**

A estimulação cerebral profunda (ECP) é um procedimento bem estabelecido para o tratamento da doença de Parkinson (DP). Embora seus mecanismos de ação não sejam claros, a melhora dos sintomas motores e a redução dos efeitos colaterais dos medicamentos são contempladas em uma proporção significativa de pacientes, com melhora da qualidade de vida.

**Objetivo:**

Investigar o impacto da ECP na qualidade de vida de pacientes em DP.

**Métodos:**

Trata-se de um estudo longitudinal retrospectivo, com coleta de dados históricos em um centro de neurocirurgia, de junho de 2019 a dezembro de 2020. A amostra foi feita por conveniência, e os questionários *Parkinson's Disease Questionnare* (PDQ-39), *Unified Parkinson's Disease Rating Scale* (UPDRS) III e IV, *Trail Making Test* e Teste de Fluência Verbal foram utilizados.

**Resultados:**

Dos dados coletados de 17 pacientes (13 ECP em núcleo subtalâmico e ECP em globo pálido interno) notou-se melhora significativa (p=0,008) no UPDRS III ao se comparar o pré-operatório sem ECP com pós-operatório com ECP, e cerca de 47,0% deles apresentaram melhora pós-cirúrgica na qualidade de vida (p=0,29). Treze pacientes conseguiram completar a parte A do *Trail Making Test* e quatro também completaram a parte B. Quase 60,0% dos pacientes obtiveram pontuação suficiente no teste semântico, enquanto apenas 11,8% obtiveram pontuação suficiente na avaliação ortográfica. Não foi possível rastrear a associação entre local do implante e desempenho.

**Conclusões:**

Melhora na qualidade de vida e na função motora foi observada na maioria dos pacientes. Apesar das limitações do estudo, a ECP beneficia fortemente uma proporção significativa de pacientes em DP quando bem indicada.

## INTRODUCTION

Parkinson's disease (PD) is a common neurodegenerative disorder characterized by motor and nonmotor symptoms that is caused by progressive degeneration of dopaminergic neurons of the substantia nigra^1,2^. Lewy bodies, in which the main component is alpha-synuclein protein, form in the substantia nigra in PD.

Deep brain stimulation (DBS) is a well-established treatment for the motor fluctuations and symptoms of PD. Although its mechanism of action is still unclear, satisfactory results are achieved when patients are properly selected. The targets most used are the subthalamic nucleus (STN), the globus pallidus pars interna (GPi) and the intermediate ventral nucleus of the thalamus (Vim)^3–5^.

Several issues need to be carefully evaluated when considering a surgical procedure. Currently, studies recommend implementation in patients over 5 years of age and under 70 years of age who have reached the maximum tolerable dose of the main drug (800 mg per day for 3 months), with motor symptoms that at some point were responsive to it. The objectives of stimulation are to alleviate the motor symptoms of the disease and reduce the adverse effects of drugs. The procedure is well indicated if an improvement of at least 30% is observed, in comparing scores from UPDRS III ON and OFF medication^6–15^.

The symptoms most responsive to stimulation are tremor, bradykinesia, stiffness and dyskinesia, but the degree of individual response is variable^6,7^. The adverse effects of DBS include axial symptoms, speech dysfunctions, cognitive or behavioral changes, dyskinesia, spontaneous muscle contractions and paresthesia, each at different degrees of intensity and incidence^7–9^.

Use of DBS is associated with an improvement in quality of life (QoL), compared with pharmacological treatment alone. However, the degree of improvement varies according to prior drug responsiveness, the predominant symptom and presence of comorbidities^10^.

Therefore, the objective of this study was to investigate the quality of life of PD patients who underwent DBS, comparing preoperative and postoperative conditions, and to assess postoperative motor and nonmotor symptoms in those patients.

## METHODS

This was a retrospective uncontrolled analytical observational longitudinal cohort study that was approved by our institution's research ethics committee. All participants signed an informed consent statement. It was conducted at the Hospital Universitário Cajuru (HUC), Curitiba, Paraná, Brazil, from June 2019 to December 2020.

### Patients

The sample was obtained according to convenience and consisted of adult patients diagnosed with PD, without cognitive problems, who were able to answer the questionnaires. All the patients underwent DBS targeted at the STN or GPi and had at least three months of follow-up after the surgical procedure. PD had been diagnosed clinically, in accordance with the presence of at least three of the following: resting tremor, bradykinesia, rigidity and postural instability.

Patients with other movement disorders and/or severe cognitive and psychiatric problems that had previously been diagnosed, those who underwent DBS targeted at the Vim and those who underwent ablative surgeries were excluded.

### Questionnaires

Preoperative questionnaires were applied during the preoperative examination, to confirm the indication for the surgery. The criterion for the postoperative evaluation was that it should be applied at least three months after the first regulation of the device, which led to variable periods after the surgery. This was due to the availability of the clinical care, as determined by the demand from patients within the public system in Brazil (Sistema Único de Saúde, SUS). In general, the examiners for the PDQ-39 and UPDRS questionnaires that were applied preoperatively were specialist doctors (neurologists and neurosurgeons). The questionnaires that were applied postoperatively were administered by the same examiners, watched by medical students who were undergoing training.

The questionnaires applied postoperatively were examined by medical students who were undergoing training and were under the supervision of specialists in the field.

An identification questionnaire was applied, which asked for the subjects’ medical record number, age, date of birth, gender, date of data collection, age at the time of diagnosis, date of implementation of the DBS, date of completion of the electrode threshold, target site, disease pattern, smoking, harmful use of alcohol, comorbidities, medications with continuous use, education, income and marital status.

To evaluate quality of life, the PDQ-39 questionnaire was applied both before and after use of DBS. This had been adapted for use in Portuguese by Health Services Research Unit (Department of Public Health and Primary Care, University of Oxford) in 2005. It consists of eight dimensions: mobility, activities of daily living, emotional wellbeing, stigma, social support, cognition, communication and body discomfort. In total, there are 39 questions with scores ranging from 0 (never) to 4 (always) that are summed for each dimension before the final score is calculated. The final score ranges from 0 (indicating no problem) to 100 (maximum problem level)^11^.

For this study, the UPDRS parts III and IV were also applied. The score for each item ranges from 0 (normality) to 4^12^. Data for the preoperative UPDRS III scale were collected from the medical records and the scale was divided into ON and OFF medication. This is also known as the levodopa challenge test, in which 50 to 100% of the levodopa dose is provided in addition to the one usually taken by the patient, in order to identify the best response. An improvement of 30–50% is generally considered necessary for the surgical procedure to be indicated. The OFF preoperative score refers to the patient's baseline state. The postoperative score, applied by the same examiner, was obtained in a state of ON stimulation and ON medication.

The Trail-Making Test has two parts: part A evaluates motor function, while part B requires mental flexibility. Thus, this test accesses the combined performance of motor and cognitive function. The time taken for application of each part of the test needs to be counted. At the end, the times are added, resulting in a final score. Patients who were unable to perform the test within 300 seconds were given a score of 300^13,14^. This test was applied only after implementation of DBS.

Lastly, an adapted verbal fluency test was applied based on a previous study. This was done only after implementation of DBS. In the first evaluation, patients were asked to say as many words as possible starting with a certain letter (e.g. B) within 60 seconds. They were then asked to say as many words as possible within a single category (e.g. animals), within 60 seconds. The score was given by the sum of the number of words (repeated words were counted only once and words that did not fit were deleted). A result consisting of 13 words or more was considered sufficient (or 9 words, in the case of illiterate patients)^13,14^.

### Statistical analysis

Frequency tables and contingency tables were created. The data distribution was determined through the Shapiro-Wilk test. Chi-square and Fisher tests were used to compare nominal and categorical data. Mann-Whitney U and unpaired t tests were used to compare numerical data. A regression analysis was performed as well, to verify the significancy of the findings through a parametric test. Both tests resulted in the same conclusion. P values < 0.05 were considered significant. All tests were calculated using the GraphPad Prism 6.0 software.

## RESULTS

**Figure 1:**
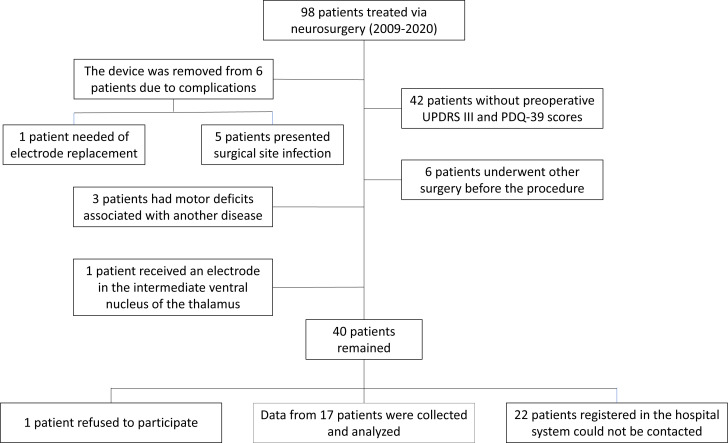
Distribution of patients during research data collection.

Between January 2009 and January 2020, 98 patients underwent DBS at our neurosurgery center. The flow diagram for patient selection can be seen in Figure 1.

From the 17-patient sample, fourteen (82.3%) were male and three (17.6%), female. The median age was 57 years, with a range from 46 to 76 years. The patients’ sociodemographic data and initial symptoms are described in Table 1. All the patients were using Levodopa and most were using one or more potentiating drugs.

**Table 1:** Sociodemographic data of the patients in this study and frequencies of initial symptoms (n=17).

Sociodemographic data		Frequency
Lifestyle habits	Smoker/ex-smoker	3 (17.6%)
	Alcoholism	1 (5.9%)
Education	Elementary/middle school incomplete	3 (17.6%)
	Elementary/middle school complete	4 (23.5%)
	High school complete	7 (41.2%)
	Higher education incomplete	1 (5.9%)
	Higher education complete	2 (11.8%)
Initial symptoms	Tremor and stiffness	5 (29.4%)
	Tremor e bradykinesia	4 (23.5%)
	Bradykinesia	4 (23.5%)
	Stiffness and bradykinesia	3 (17.6%)
	Bradykinesia and postural instability	1 (5.9%)

The patients had, on average, been diagnosed approximately 12.1±4.2 years earlier when they underwent the first DBS procedure and 14.6 years had passed since receiving the diagnosis, at the time of data collection. The median age at the first surgery was 55 years. The STN was the implementation site for 13 patients (76.5%), while GPi was chosen for four patients (23.5%). Twelve patients (70.6%) underwent operations bilaterally. Among the five patients with unilateral implementation, three (60.0%) received DBS in the STN and two (40.0%) in the GPi.

Nine patients (52.9%) had the tremor-dominant subtype. Among these, five (44.4%) showed improvements in motor function and quality of life, three (33.3%) had an improvement only in motor function and one (11.1%) improved only on the QoL scale. The most prominent symptoms before surgery are described in Table 2.

**Table 2:** Relationship between the main preoperative symptoms and the stimulation site chosen.

Stimulation site and symptoms		Frequency
STN (n=13) (76.5%)	Tremor	3 (27.3%)
	Dyskinesia	3 (27.3%)
	Bradykinesia and tremor	2 (15.4%)
	Stiffness	2 (15.4%)
	Dyskinesia and tremor	1 (7.7%)
	Bradykinesia	1 (7.7%)
	Stiffness and bradykinesia	1 (7.7%)
GPI (n=4) (23.5%)	Dyskinesia	4 (100%)

STN: subthalamic nucleus; GPI: globus pallidus pars interna.

**Figure 2:**
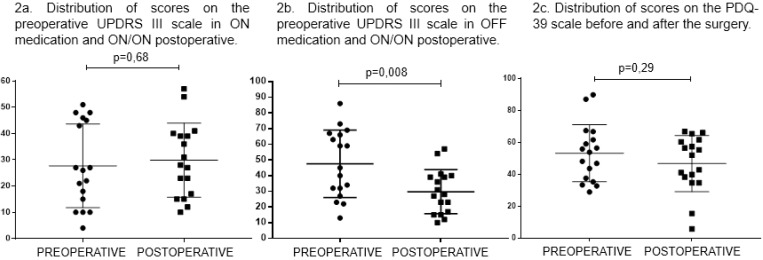
Distribution of Unified Parkinson's Disease Rating Scale III and Parkinson's Disease Questionnaire scores.

Comparing the results from the preoperative UPDRS III scale (OFF medication) and from the postoperative scale (ON medication and ON DBS), thirteen patients (76.5%) had improved scores. The mean improvement in this comparison was 49.6% (±20.4%). Among these 13 patients with improvements in relation to the preoperative OFF score, ten (76.9%) also improved in relation to the levodopa challenge test (ON medication), performed preoperatively. The mean improvement in this case was 29.3% (±15.6%). For nine (69.2%) of the 13 patients with motor improvement, the evaluation was made one year or more after the last surgical procedure. Two (50.0%) of the four patients without improvement on the UPDRS III scale had been diagnosed with PD more than 15 years earlier. Eight (61.5%) of the 13 patients with motor improvement were under 60 years of age. The distribution of scores can be observed in Figure 2a, 2b and 2c.

Regarding the assessment of quality of life through the PDQ-39, eight patients (47%) reported having improvements in quality of life after surgery, by an average of 48.3% (±30.3%), although this change was not statistically significant (p=0.29). Six (75%) of the eight patients with improved quality of life were less than 60 years of age. Three patients (37.5%) with unilateral electrode implantation had an average improvement in the PDQ-39 of 47.2% (±40.8%).

The individual evaluation of the domains in the PDQ-39 revealed that the domain that benefited the most was wellbeing, in which 68.75% of the patients showed improvements in relation to the presurgical scale. Furthermore, 31.25% showed improvement in mobility, and all of these patients also showed improvement in wellbeing and were under 60 years old. Out of the total number of patients under 60 years old, 55.56% showed improvements in both mobility and wellbeing. There were eight patients with worsening cognition, among whom 62.5% were over 60 years old, while 71.43% of the seven patients with improved cognition were under 60 years old. However, these results were not statistically significant. It is important to note that one of the patients included in the present study did not have presurgical data relating to each domain separately and was excluded from the individual analyses on the PDQ-39 domains. The distribution of scores on the PDQ-39 scale can be seen in Graph 1C. Most patients, when subjectively questioned, reported having substantial improvements in quality of life and motor function.

Among the 17 patients, five (29.4%) had had less than one year of follow-up after undergoing DBS, at the time of data collection. There was no relationship between a length of follow-up of less than one year and more promising results regarding motor function and quality of life.

Among the 13 patients with STN stimulation, twelve (92.3%) had improvements in UPDRS III score in relation to the preoperative OFF score, and six (46.1%) also showed improvements in the PDQ-39 score. Three (75.0%) of the four patients with GPi stimulation did not have any improvement in motor function and two (50.0%) reported having an improvement in quality of life. All the patients with postoperative improvement in relation to the preoperative UPDRS III ON had bilateral electrode implantation.

Part IV of the UPDRS was evaluated only in the postoperative period. The distribution of patients in different states of disease according to age and time since diagnosis can be seen in Table 3.

**Table 3:** Motor staging (Unified Parkinson's Disease Rating Scale IV) in relation to age and time since diagnosis (n=17).

Hoehn & Yahr stage			Age (years)*	Time since diagnosis (years)
0	No illness	-	-	-
1	Unilateral disease	1 (5.9%)	53	13
2	Bilateral disease without balance deficit	5 (29.4%)	54 (49–65)	14 (9–18)
3	Mild to moderate bilateral disease, some postural instability, but there is an ability to live independently	10 (58.8%)	59 (46–76)	14.8 (8–22)
4	Severe disability, but still able to walk or stand without help	1 (5.9%)	71	21
5	Confined to bed or wheelchair	-	-	-

* In the “age” column, the number outside the parentheses indicates the median age, and the numbers inside the parentheses indicate the minimum and maximum age.

Regarding the Trail-Making Test, thirteen patients (76.5%) completed part A, and four of these (30.8%) completed part B. Four patients (23.5%) did not complete part A and did not proceed to the second part of the test. Two patients (50.0%) who completed both parts of the test had undergone GPi stimulation and two (50.0%) had undergone STN stimulation. The four patients (10.8%) who completed the test had a subtype of disease other than dominant tremor.

The distribution of the patients in the parts of the verbal fluency test according to the stimulation site is shown in Table 4.

**Table 4:** Semantic and phonemic verbal fluency test and DBS sites (n=17).

Verbal fluency test			
Semantic		Phonemic	
Sufficient	10 (58.8%)	Sufficient	2 (11.8%)
STN	7 (70%)	STN	1 (50%)
GPI	3 (30%)	GPI	1 (50%)
Insufficient	7 (41.2%)	Insufficient	15 (88.2%)
STN	6 (85.7%)	STN	12 (80%)
GPI	1 (14.3%)	GPI	3 (20%)

STN=13 patients; GPI=4 patients. STN: subthalamic nucleus; GPI: globus pallidus pars interna.

## DISCUSSION

Although DBS is a surgical procedure with a great impact on QoL, it is not clearly demonstrated in the literature how much it interferes in the most diverse areas of life of patients with PD. The sample obtained in our study was equivalent to more than a third of the population with potential for analysis. Our research reiterated some results already reported by others^3,16–20^, but it also came up with other data, thus raising questions for possible future investigation.

In this study, a significant improvement in general motor function compared with the presurgical OFF period could be seen. Nevertheless, this cannot indicate any definitive conclusion regarding the efficacy of the method, considering that the comparison was with patients who were ON DBS and ON medication. Among the patients without any improvement in motor function, half presented disease at a more advanced stage.

The preoperative levodopa challenge test requires at least 30–50% improvement of motor symptoms in relation to the OFF phase, without medication. Furthermore, the indication should be individualized and should include assessment of nonmotor symptoms^6,21,22^. The presence of comorbidities such as frank dementia or severe cognitive dysfunction formally contraindicate stimulation, as there will be no benefit from treatment^7,9^. If the criteria are met, there is a higher likelihood of favorable results from stimulation^23^.

The clinical worsening that was noticed in a few patients after DBS may be attributed to the disease progression itself. However, it is usually possible to adjust the stimulation patterns, with at least partial improvement of the condition^9,19,24,25^.

In the present study, no statistically significant improvement in QoL was observed through the PDQ-39, and bilateral stimulation did not reveal any greater impact, as had been reported by two other studies^26,27^. Despite the objective results, there was a substantial improvement in QoL according to the subjective perception of most patients. These assessments were made based on the patient's report of perceived improvement or worsening of the clinical condition.

Objective scales for quality-of-life assessment are widely used, but some studies have also found no correlation between the scores obtained through the objective questionnaire and the overall satisfaction subjectively reported by patients^16,17^. Frizon et al. proposed three variables capable of predicting improvement in up to 81.4% of the cases: PDQ-39 preoperatively, percentage of improvement of UPDRS-III after levodopa use and years since the onset of symptoms. According to the literature, worse preoperative PDQ-39 scores and high percentage of medication response are predictors of greater chance of improvement in quality of life^18,23,24,28^.

Moreover, in large meta-analyses, an average improvement of 34.5% in the quality of life of patients with bilateral stimulation assessed through the PDQ-39 was reported, with a range from 14 to 62%. The average improvement through bilateral stimulation in the present study was slightly higher (41%; SD 27.5%). Few studies have been conducted regarding unilateral stimulation. The study by Slowinski et al., 2007, showed a mean improvement of 15% among patients with a unilateral electrode, while the study by Frizon et al. showed a median improvement of 34.6% among patients with unilateral stimulation, compared with an improvement of 44.1% among those with bilateral stimulation^18,20^.

It was not possible to observe any influence from more recent surgeries (less than one year) on motor function and quality of life in most of the patients. This was contrary to what most studies have shown, i.e. that the greatest benefit of therapy was within the first 6 to 12 months after surgery. Some other studies have indicated differences in motor outcomes, with worsening as the time elapsed after the procedure increased. However, those studies used longer intervals (five years) as the cutoff because it was believed that the main effects of STN-DBS could last for up to five years. The effects of GPi-DBS would last for a slightly shorter time, independently of the onset of PD. Motor fluctuations, dyskinesia and activities of daily living should also be improved through stimulation, although a decline in the benefit over the years has been identified^19,25^.

One group reported rates of improvement in UPDRS III score of 45% over five years and 42% over ≥9 years, which were similar to the rates observed in the present study. In addition, there is evidence that some patients can expect improvement even after 10 years of stimulation, but with reductions in the UPDRS-III score of 25.3%^19^.

Compared with STN, GPi stimulation does not allow significant reductions in medication intake. However, it has a direct effect on inhibition of drug-related dyskinesias, with a reduction in incidence of up to 80%. Thus, GPi-DBS enables increases in daily dosage with fewer concomitant side effects, and also improvement of nonmotor symptoms that are responsive to dopaminergic medication. According to Chao et al., the main advantage of DBS, regardless of the implementation site, is the potential for adjusting the stimulator at any time after surgery in order to maximize benefits and minimize adverse effects^3,4,22,29^.

Studies have indicated there is no significant difference in UPDRS results between implementation sites, except for the slight improvement of stiffness and axial symptoms seen with GPi-DBS^15,23,24,26^. However, we observed that STN-DBS produced a more significant improvement of symptoms during the OFF medication period. A previous study showed that there was an improvement in UPDRS-III of around 41% under these circumstances^30^. Thus, STN-DBS would be better indicated for patients with low levodopa tolerance, in order to enable greater postoperative dose reduction^3,7,15,24^.

Although most studies have suggested that GPi is the most appropriate site, considering cognitive and neuropsychiatric symptoms, discrepancies in the results still exist among different centers. Authors who obtained more favorable outcomes with GPi-DBS used higher doses of dopaminergic medication, and this factor may explain this finding^23,24,28^.

The results found in the current study emphasized the deterioration of executive function. This was characterized by increased time taken to perform the Trail-Making Test, part B. Therefore, as noticed in previous studies, a possible relationship with older age and the natural progression of the disease was identified. Nonetheless, despite the hypotheses, the impact of DBS on executive function is not yet well established, and existing studies have demonstrated discordant results. Some cognitive changes observed after brain stimulation can be evaluated through the Trail-Making Test. In the study by Sáez-Zea et al. there was an increase in the time taken to perform part B of the test, both among patients with STN-DBS and among those treated only with pharmacotherapy. However, it is noteworthy that there was a statistically significant relationship between older age and longer time taken to perform this part of the test. Both the neuropsychological and the motor changes observed after surgery vary according to disease subtype, lead position, distribution of electric current and changes in drug therapy^28,30^.

Semantic and phonemic verbal fluency were found to have become impaired after surgery in our patients. Phonemics were worsened regardless of implantation site, while semantics became more impaired in patients with STN-DBS. This was possibly due to decreased activation of the lower prefrontal and temporal cortex of the left cerebral hemisphere. Longer follow-up (more than one year), education level and age did not interfere with the outcomes, which differed from the results obtained in the study by Olchik et al., where these factors were associated with worse cognitive performance^2^.

Speech disorders occur in up to 89% of individuals with PD, regardless of age and length of time with the disease^25^. Although some studies have shown that DBS helps to improve speech mechanisms, most have demonstrated that patients with STN-DBS present deteriorated speech intelligibility, and this procedure has also been associated with negative impacts on intonation, rhythm and articulation, and hypophony has been found to be the most frequent effect^25,28,31,32^. In patients who underwent GPi-DBS, speech deterioration has not been so commonly reported. However, its effects on speech have been less studied than those of STN-DBS^31,32^. Although phonemic verbal fluency was more affected, semantics were also impaired^25,28,30^.

The limitations of this study were its small sample size and cross-sectional design; strict inclusion criteria; and the impossibility of expanding the face-to-face evaluation due to paralyzation of outpatient activities caused by the Covid-19 pandemic. Further research to understand QoL after DBS to treat PD is still required.

In conclusion, both quality of life and motor function presented improvements through DBS, although quality-of-life improvements were not statistically significant. Nonmotor symptoms did not present a favorable outcome in most patients. Despite the favorable results achieved through DBS for treating PD, further research is still required.
